# Age, but Not Amyloidosis, Induced Changes in Global Levels of Histone Modifications in Susceptible and Disease-Resistant Neurons in Alzheimer’s Disease Model Mice

**DOI:** 10.3389/fnagi.2019.00068

**Published:** 2019-04-03

**Authors:** Marcus Dyer, Andrew J. Phipps, Stanislaw Mitew, Phillippa C. Taberlay, Adele Woodhouse

**Affiliations:** ^1^Wicking Dementia Research and Education Centre, College of Health and Medicine, University of Tasmania, Hobart, TAS, Australia; ^2^Menzies Institute for Medical Research, College of Health and Medicine, University of Tasmania, Hobart, TAS, Australia; ^3^Duke-NUS Medical School, National University of Singapore, Singapore, Singapore; ^4^School of Medicine, College of Health and Medicine, University of Tasmania, Hobart, TAS, Australia

**Keywords:** epigenetics, H3K4me3, H4K27ac, H3K27me3, calretinin, neurofilament triplet proteins

## Abstract

There is increasing interest in the role of epigenetic alterations in Alzheimer’s disease (AD). The epigenome of every cell type is distinct, yet data regarding epigenetic change in specific cell types in aging and AD is limited. We investigated histone tail modifications in neuronal subtypes in wild-type and APP/PS1 mice at 3 (pre-pathology), 6 (pathology-onset) and 12 (pathology-rich) months of age. In neurofilament (NF)-positive pyramidal neurons (vulnerable to AD pathology), and in calretinin-labeled interneurons (resistant to AD pathology) there were no global alterations in histone 3 lysine 4 trimethylation (H3K4me3), histone 3 lysine 27 acetylation (H3K27ac) or histone 3 lysine 27 trimethylation (H3K27me3) in APP/PS1 compared to wild-type mice at any age. Interestingly, age-related changes in the presence of H3K27ac and H3K27me3 were detected in NF-labeled pyramidal neurons and calretinin-positive interneurons, respectively. These data suggest that the global levels of histone modifications change with age, whilst amyloid plaque deposition and its sequelae do not result in global alterations of H3K4me3, H3K27ac and H3K27me3 in NF-positive pyramidal neurons or calretinin-labeled interneurons.

## Introduction

Alzheimer’s disease (AD) is a progressive neurodegenerative disease characterized by the accumulation of intracellular tau in neurofibrillary tangles and neuropil threads, and the deposition of insoluble extracellular β-amyloid (Aβ) plaques associated with dystrophic neurites and synapse loss (Braak and Braak, 1991; Thal et al., 2002). A specific subset of excitatory pyramidal neurons that express neurofilament (NF) proteins are particularly susceptible to neurofibrillary tangle formation, dystrophic neurite formation, synapse loss, degeneration and death in AD (Hof et al., 1990; Thangavel et al., 2009; Mitew et al., 2013a,b). In comparison, inhibitory interneurons are relatively resistant to AD pathology (Hof and Morrison, 1991; Hof et al., 1993; Mitew et al., 2013a,b).

AD has a complex etiology including many lifestyle-related risk factors, the greatest of which is increasing age. As the interface between our genes and the environment, the epigenome is well positioned to provide a mechanistic link in the onset and progression of AD. There is increasing evidence that epigenetic alterations occur in the aging brain and AD (Gräff et al., 2012; Chouliaras et al., 2013; Lister et al., 2013; Ziller et al., 2013; Coppieters et al., 2014; De Jager et al., 2014; Lunnon et al., 2014; Benito et al., 2015; Gjoneska et al., 2015; Mastroeni et al., 2015; Gasparoni et al., 2018; Nativio et al., 2018), and that epigenetic changes occur early in AD and change in tandem with disease progression (De Jager et al., 2014; Lunnon et al., 2014; Sanchez-Mut et al., 2014; Gasparoni et al., 2018). However, most studies analyze brain homogenate and little is known about the epigenetic alterations that occur in specific cell types in AD.

The epigenome is a highly interactive network of chemical moieties including DNA methylation and histone modifications that governs the packaging of DNA into chromatin within the nucleus. The basic repeating unit of chromatin is the nucleosome, which is comprised of an octamer of alkaline histone proteins, typically canonical H2A, H2B, H3 and H4, which form dimers and exhibit protruding N-terminal tails that are subject to post-translational modification (Phillips and Johns, 1965; Kornberg, 1974; Thomas and Kornberg, 1975). The type of histone proteins present in a nucleosome and the various post-translational modifications (including methylation and acetylation) determine the affinity with which DNA is bound in the nucleosome, serve to recruit histone reader and modifier proteins and thus, modulate the accessibility of DNA for transcription (Allfrey and Mirsky, 1964; Allfrey et al., 1964). Thus, different histone modifications mark signatures are associated regions of DNA that are accessible and active, or compact and repressed (Barski et al., 2007; Heintzman et al., 2007). For instance, trimethylation of histone 3 at lysine 4 (H3K4me3) marks active promoters, H3K27ac marks active promoters and enhancers, while H3K27me3 marks repressed promoters (Cao et al., 2002; Bernstein et al., 2005; Creyghton et al., 2010).

The epigenetic signature and gene expression profile of each cell type is unique and there is increasing interest in identifying cell-type specific epigenetic phenotypes in the brain. The vast majority of studies investigating epigenetic alteration in AD have used whole brain homogenate containing both neurons and glia (Gräff et al., 2012; Chouliaras et al., 2013; Coppieters et al., 2014; De Jager et al., 2014; Lunnon et al., 2014; Sanchez-Mut et al., 2014; Gjoneska et al., 2015; Nativio et al., 2018), likely given the technical complexity of isolating individual cell types from brain tissue. Several recent studies have highlighted the importance of examining purified neurons and glia; as neurons and glia exhibit distinct epigenetic profiles that responded differently to amyloid plaque pathology and therapeutic interventions (Kozlenkov et al., 2014; Benito et al., 2015; Gasparoni et al., 2018; Zhao et al., 2018). Furthermore, diverse and highly distinctive epigenomic signatures have been detected in neuronal subsets, including excitatory neurons and inhibitory interneurons (Mo et al., 2015; Kozlenkov et al., 2016; Luo et al., 2017). Examining epigenetic marks in subpopulations of neurons is particularly important in AD, as NF-positive pyramidal neurons are particularly susceptible to dysfunction, degeneration and death in AD (Hof et al., 1990; Thangavel et al., 2009; Mitew et al., 2013a,b).

While numerous studies have identified DNA methylation alterations in aging and AD (Chouliaras et al., 2013; Lister et al., 2013; Sanchez-Mut et al., 2013, 2014; Ziller et al., 2013; Coppieters et al., 2014; De Jager et al., 2014; Lunnon et al., 2014; Gasparoni et al., 2018), there are relatively few studies that have investigated histone modifications in AD and in aged neurons (Benito et al., 2015; Gjoneska et al., 2015; Mastroeni et al., 2015; Narayan et al., 2015; Nativio et al., 2018). To date, dysregulation of the H4K16ac, H3K4me3, H4K12ac and H3K27ac histone marks have been reported in human AD and in mouse models of AD. In the temporal gyrus of AD cases, there was a dysregulation of H4K16ac peaks in AD including both gains and losses that were associated with pathways including signaling cascades, apoptosis/cell death and cell communication (Nativio et al., 2018). Similarly, significant alterations in H3K4me3 in promotor regions and H3K27ac at enhancer regions (including gains and losses) associated with increased immune system processes and decreased synaptic transmission, learning and memory were identified in the hippocampus of the CK-p25 mouse model of neurodegeneration (Gjoneska et al., 2015). However, in the hippocampus and medial temporal gyrus of human AD cases, there was no alteration in overall H3K4me3 protein levels detected, however a decrease in nuclear H3K4me3 concomitant with increased abnormal cytoplasmic H3K4me3 was observed (Mastroeni et al., 2015). Finally, a study by Benito et al. (2015) reported a decrease in H4K12ac at the transcriptional start site of neuronal and non-neuronal cells in transgenic (TG) AD model mice that was associated with the downregulation of synaptic plasticity genes in the hippocampus. Notably, there are only two published articles to date (Benito et al., 2015; Mastroeni et al., 2015) examining histone mark alterations specifically in neurons in AD.

We investigate the histone marks H3K4me3, H3K27ac and H3K27me3 in APP/PS1 and wild-type mice at 3, 6 and 12 months of age, representing pre-pathology, pathology onset and pathology-rich time points in this mouse model. APP/PS1 mice are a well-characterized mouse model of amyloid deposition that develop Aβ plaques, plaque-associated dystrophic neurites, synapse loss and memory deficits with age (Jankowsky et al., 2004; Garcia-Alloza et al., 2006; Kilgore et al., 2010; Volianskis et al., 2010; Mitew et al., 2013a,b). Differences in the vulnerability of NF-labeled pyramidal neurons and calretinin-positive interneurons have also been demonstrated in APP/PS1 mice. For example, twice the proportion of NF-labeled neurites in the vicinity of Aβ plaques exhibited neuritic dystrophy compared to calretinin-labeled neurites in APP/PS1 mice (Mitew et al., 2013b), and loss of excitatory, but not inhibitory, presynaptic puncta was observed adjacent to plaques in APP/PS1 mice (Mitew et al., 2013a). Thus, we examine histone alterations in disease-resistant calretinin-labeled interneurons and the vulnerable NF-positive pyramidal neurons for the first time in APP/PS1 mice.

## Materials and Methods

### Mouse Cohort

All experiments were approved by the Animal Ethics Committee of the University of Tasmania (#A12780/A15170) and were undertaken according to the Australian Code of Practice for the Care and Use of Animals for Scientific Purposes. TG APP/PS1 mice expressing APP with the familial Swedish mutation (KM670/671NL) and mutant PS1 (PSENdE9; APP/PS1) under the prion protein promoter on a C57BL/6 background strain (Jankowsky et al., 2004). Amyloid plaques and memory deficits appear at 6 months of age (Jankowsky et al., 2004; Kilgore et al., 2010) and plaque load increases up to 12 months of age (Garcia-Alloza et al., 2006). By 12 months of age APP/PS1 mice exhibit spatial memory deficits, and significant plaque-associated dystrophic neurite pathology and excitatory synapse loss of age (Volianskis et al., 2010; Mitew et al., 2013a,b). Neuronal loss has been reported adjacent to amyloid plaques in 8–10-month-old APP/PS1 mice (Jackson et al., 2016), but does not recapitulate the extensive neuronal loss observed in human AD.

Male APP/PS1 mice aged 3 months (*n* = 10), 6 months (*n* = 10) and 12 months (*n* = 9) and age matched C57BL/6 wild-type mice (*n* = 10 of each age) were used in this study. All animals were housed in standard laboratory conditions (25°, 12 h light/dark cycle) with *ad libitum* access to food and water.

Genotyping of wild-type and APP/PS1 mice was performed as previously (Fernandez-Martos et al., 2015). Briefly, mouse tail genomic DNA was extracted using the Extract-N-Amp Tissue PCR Kits (Sigma-Aldrich) and then, standard polymerase chain reaction (PCR) analysis was performed using MyTaq^TM^ DNA Polymerase (Bioline) with primers to mouse presenilin-1 (For: 5′-AATAGAGAACGGCAGGAGCA-3′; Rev: 5′-GCCATGAGGGCACTAATCAT-3′) and interleukin 2 (For: 5′-CTAGGCCACAGAATTGAAAGATCT-3′; Rev: 5′-GTAGGTGGAAATTCTAGCATCATCC-3′) as an internal control. Reactions (10 μL) consisted of 1 μL DNA template and primers at a final concentration of 0.5 μM. Cycling was performed as follows: hold at 94°C for 2 min followed by 10 cycles of [94°C for 20 s 65°C (decreasing by 0.5°C per cycle) for 15 s and 68°C for 10 s] followed by 28 cycles of (94°C for 15 s 60°C for 15 s and 72°C for 10 s) followed by a hold at 72°C for 2 min and then at 10°C. Amplified PCR products were then visualized by agarose gel electrophoresis to determine genotypes. In addition, one tissue section from each mouse used in this study was stained with Thioflavine S and visualized on an Olympus BX50 fluorescent microscope to confirm the genotyping data. To perform Thioflavine S staining, tissue sections were incubated in 0.125% Thioflavine S dissolved in a solution of 40% ethanol and 60% 0.01M phosphate buffered saline (PBS) for 3 min on an orbital shaker at room temperature, tissue sections were then washed twice in a 50:50 solution of ethoanol:0.01M PBS for 1 min each, followed by three washes in 0.01M PBS for 10 min each on an orbital shaker.

### Immunohistochemistry

Mice were anaesthetized with 150 μL of 60mg/mL sodium pentobarbitone and transcardially perfused with 4% paraformaldehyde in 0.1M PBS. Brain tissue was cryoprotected (18% then 30% sucrose in PBS) and 40 μm serial coronal sections were cut on a cryostat (Leica CM1850 cryostat with O.C.T compound; ProSciTech, Australia).

Double-label immunohistochemistry was performed to assess the alterations in the histone marks H3K4me3, H3K27me3 and H3K27ac in NF-containing pyramidal neurons (using anti-SMI32) and in calretinin-positive interneurons (using an anti-calretinin antibody; Table 1). One coronal section between bregma 1.53 mm and −1.56 mm containing the S1/S2 somatosensory cortex and the M1/M2 motor cortex from each mouse was immunolabeled with each antibody combination. The analysis was performed in the S1/S2 somatosensory cortex and the M1/M2 motor cortex as significant Aβ plaque deposition, dystrophic neurite pathology and plaque-associated synaptic loss occur in these brain regions in APP/PS1 mice (Mitew et al., 2013a; Fernandez-Martos et al., 2015). Double-label immunohistochemistry and DAPI staining were performed as previously described (Phipps et al., 2016) with fluorescent secondary antibodies (1:1,000) used for visualization (Table 2). To assess Aβ plaque load in 3- (*n* = 9), 6 (*n* = 10) and 12- (*n* = 8) month-old APP/PS1 mice, one tissue section between bregma 1.53 mm and −1.56 mm containing the S1/S2 somatosensory cortex and the M1/M2 motor cortex from each mouse was subjected to formic acid antigen retrieval and Aβ labeling with the anti-6E10 antibody was performed as previously (Phipps et al., 2016). To assess presynaptic bouton density one tissue section containing the somatosensory cortex (S1) from 2- and 12-month-old wild-type mice (*n* = 5) were immunolabeled with synaptophysin, vesicular glutamate transporter 1 (VGlut1) or vesicular GABA transporter (VGAT, Table 1) as previously described (Mitew et al., 2013a). The dilution of each primary antibody was optimized and omitting primary antibodies eliminated all immunosignal. For information regarding the specificity of primary antibodies used in this study please refer to the Supplementary Material.

**Table 1:** Primary antibodies.

Name	Epitope	Manufacturer	Catalogue number	Host species	Concentration
H3K4me3	Histone 3, Lysine 4 trimethyl	Active Motif (USA)	39160	Rabbit	1:1,000
H3K27me3	Histone 3, Lysine 27 trimethyl	Millipore (USA)	07–449	Rabbit	1:1,000
H3K27ac	Histone 3, Lysine 27 acetylated	Active Motif (USA)	39134	Rabbit	1:1,000
SMI-32	NF-M and NF-H non-phosphorylated	Biolegend (USA)	SMI-32P	Mouse	1:500
Calretinin	Calretinin-22K	Swant (Switzerland)	6B3	Mouse	1:250
6E10	Purified β-amyloid 1–16	Covance (USA)	SIG-39320	Mouse	1:1,000
Synaptophysin	Synthetic full-length human synaptophysin	Millipore (USA)	AB9272	Rabbit	1:200
VGlut1	Recombinant protein aa 456–560 of VGlut1	Synaptic Systems (Germany)	135 303	Rabbit	1:500
VGAT	Synthetic peptide aa 75–87 of rat VGAT	Synaptic Systems (Germany)	131 003	Rabbit	1:500

**Table 2:** Secondary antibodies.

Fluorophore	Manufacturer	Catalogue number	Reactivity	Species
Alexa Fluor 594	Invitrogen (USA)	A-11023	Anti-rabbit IgG	Goat
Alexa Fluor 488	Invitrogen (USA)	A-11029	Anti-mouse IgG	Goat

### Microscopy and Analysis

All image analysis was performed blinded to mouse genotype and age. Full coronal tissue sections were imaged on an Olympus VS120 slide scanner (U-HGLGPS light source, Olympus, USA) at 20× optical zoom. Autofocusing was optimized for either SMI-32 or calretinin labeling, an exposure of 50 ms was used to capture images of SMI-32 and calretinin labeling, and an exposure of 25 ms was used to capture images of H3K4me3, H3K27ac and H3K27me3 labeling and DAPI staining.

To determine whether H3K4me3, H3K27me3 and H3K27ac were altered in all cortical nuclei across disease progression in APP/PS1 mice, the percentage of nuclei co-localized with each histone mark was quantitated in neocortical layers 1–6 and in layer 2/3. In each tissue section, two regions of interest (ROIs) encompassing layers 1–6 and layers 2/3 of the neocortex of one hemisphere from the longitudinal fissure to the rhinal fissure were selected. Both DAPI stained nuclei and H3K4me3, H3K27me3 and H3K27ac-labeled nuclei were quantitated in each ROI in Image J with the nucleus counter plugin [(Szabo and Gulya, 2013; Ioannou et al., 2014); see Supplementary Figure S1].

To assess alterations in H3K4me3, H3K27ac and H3K27me3, co-localization in NF-positive pyramidal neurons and calretinin-labeled interneurons in the cortex of APP/PS1 mice, 50 SMI32-positive pyramidal neurons and 50 calretinin-labeled interneurons were systematically counted in layers 2/3 of the M1/M2 motor cortex and S1/S2 somatosensory cortex in one tissue section from each mouse.

To determine Aβ plaque load tissue sections were imaged on an Olympus VS120 slide scanner (U-HGLGPS light source, Olympus, USA) at 20× optical zoom. Autofocusing was optimized for Aβ labeling and an exposure of 40 ms was used. A region of interest was drawn to contain all of one hemisphere of the neocortex dorsal to the rhinal fissure and thresholded to the labeled signal (FIJI ImageJ). The percentage area occupied by Aβ was calculated by dividing the total area occupied by plaques by the total area analyzed as previously (Fernandez-Martos et al., 2015).

To quantitate presynaptic bouton density regions of interest were imaged in layer 2/3 of the somatosensory cortex (S1) from each tissue section (3–5 regions of interest/section) immunolabeled with synaptophysin, VGlut1 and VGAT. Image acquisition and synaptic puncta analysis were performed as previously described (Mitew et al., 2013a). Briefly, z-stacks of each region of interest were acquired on a Zeiss LSM510 confocal microscope (Zen software, Germany), each z-stack was merged into one image, images were automatically thresholded, processed for Gaussian blurring and watershed prior to particle analysis being performed to quantitate synaptic puncta (ImageJ; Mitew et al., 2013a).

Data analysis was performed in SigmaPlot version 11.0 (Systat Software Inc., Chicago, IL, USA). One way analysis of variance (ANOVA) with *post hoc* Holm-Sidak, or a Kruskal-Wallis one way analysis of ranks with *post hoc* Holm-Sidak were employed when comparing more than two experimental groups depending on the distribution (normality, variance) of the data. A two-tailed type 3 Student’s *t*-test was used when comparing two experimental groups. The statistical test used for each dataset is detailed in the Results section. A *p*-value of <0.05 was used as the definition of statistical significance and all data are represented as the mean ± the standard error of the mean (SEM).

### Images for Figures

Representative images for figures were taken on an Olympus VS120 slide scanner (U-HGLGPS light source, Olympus, USA) or a Perkin Elmer spinning disk confocal microscope (Zeiss LSM710 laser scanning microscope) and Perkin Elmer’s velocity software (Perkin Elmer). Brightness and contrast adjustments were made consistently across all images to enhance the clarity of images with Image-J and Adobe Photoshop CS6 (Adobe, CA, USA). Graphs were produced in GraphPad Prism 6 (GraphPad Software Inc., San Diego, CA, USA).

## Results

### No Difference in the Percentage of Nuclei Labeled With H3K4me3, H3K27me3 or H3K27ac in the Cortex of APP/PS1 Mice Compared to Wild-Type Mice

**Figure 1:**
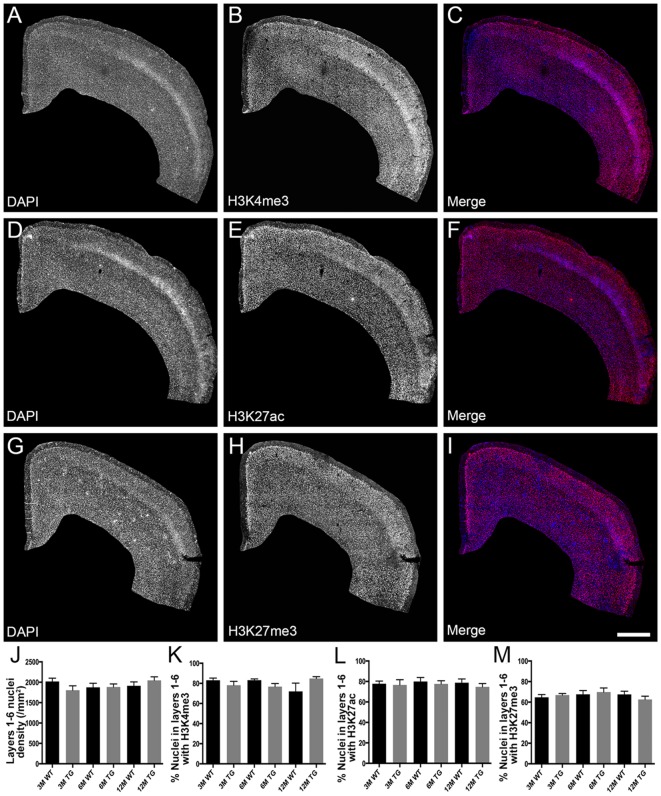
No difference in the percentage of nuclei immunolabeled with H3K4me3, H3K27me3 or H3K27ac in layers 1-6 of the cortex in APP/PS1 mice compared to wild-type mice. Representative images of coronal sections stained with DAPI (blue, **A,C,D,F,G,I**) and immunolabeled for H3K4me3 (red, **B,C**), H3K27ac (red, **E,F**) and H3K27me3 (red, **H,I**) in a 3 month old APP/PS1 mouse **(A–C)**, a 12 month old APP/PS1 mouse **(D–F)** and a 3 month old wild type (WT) mouse **(G–I)**. **(J)** Bar graph displaying the density of DAPI stained nuclei in layers 1-6 of 3-month (3M), 6-month (6M) and 12-month (12M) old WT and APP/PS1 transgenic (TG) mice. Bar graphs showing the percentage of nuclei co-localized with H3K4me3 **(K)**, H3K27ac **(L)** and H3K27me3 **(M)** at 3, 6 and 12 months of age in APP/PS1 and wild-type mice. All data presented as mean ± standard error of the mean (SEM). Scale bar = 400 μm.

**Figure 2:**
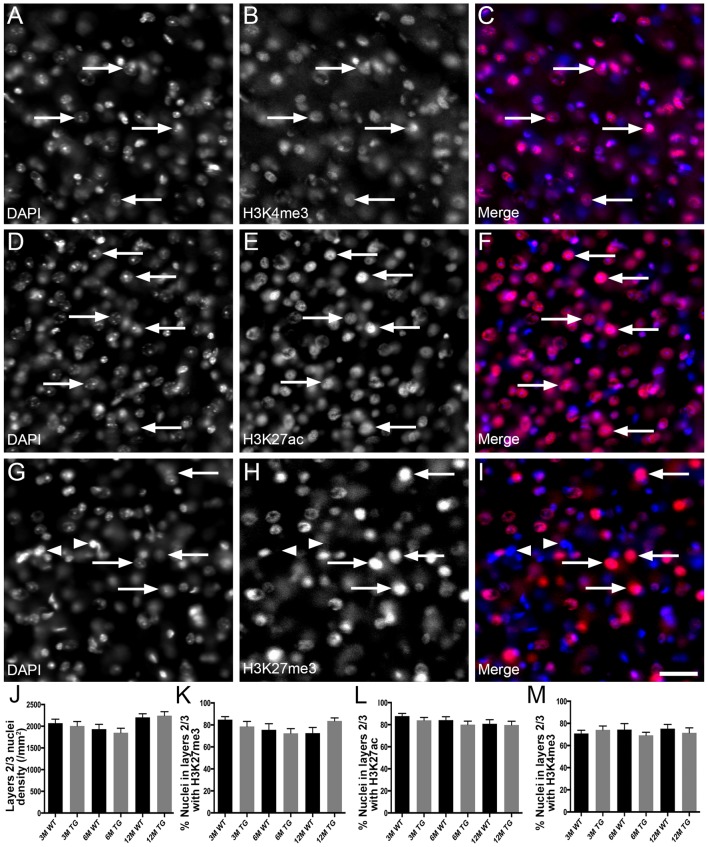
No change in the proportion of nuclei in layer 2/3 of the cortex co-localized with H3K4me3, H3K27me3 or H3K27ac in APP/PS1 mice vs. wild-type mice. Example images of areas layer 2/3 stained for DAPI (blue, **A,C,D,F,G,I**) and immunolabeled for H3K4me3 (red, **B,C**), H3K27ac (red, **E,F**) and H3K27me3 (red, **H,I**) in a 6 month old WT mouse **(A–C)**, a 6 month old APP/PS1 mouse **(D–F)** and a 12 month old WT mouse **(G–I)**. Note nuclei positive (arrows) and negative (arrowheads) for histone mark immunoreactivity. **(J)** Bar graph displaying the density of nuclei in layer 2/3 of 3-month (3M), 6-month (6M) and 12-month (12M) WT mice and TG APP/PS1 mice. Bar graphs showing the percentage of nuclei immunoreactive for H3K4me3 **(K)**, H3K27ac **(L)** and H3K27ac **(M)**. All data presented as mean ± SEM. Scale bar =50 μm.

H3K4me3 and H3K27ac labeled approximately 79% of cortical nuclei and H3K27me3 was present in approximately 67% of cortical nuclei (Figure 1, Supplementary Table S1). There was no difference in the density of DAPI-stained nuclei in layers 1–6 or layers 2/3 of the cortex due to age or APP/PS1 transgene expression (layers 1–6, Kruskal-Wallis one way ANOVA on ranks, Figure 1J; layers 2/3, one way ANOVA, *p* > 0.05; Figure 2J). There were no alterations to the percentage of nuclei immunolabeled with H3K4me3, H3K27me3 or H3K27ac cortical layers 1–6 (H3K4me3, Kruskal-Wallis one way ANOVA on ranks; H3K27me3 and H3K27ac, one way ANOVA; Figure 1) or in layers 2/3 (one way ANOVA, Figure 2) due to the age or genotype. The Aβ plaque load was quantitated, and as expected progressively increased with age in 3- (0.21 ± 0.1%), 6- (1.5 ± 0.5%) and 12- (15.3 ± 3.1%) month-old APP/PS1 mice.

### No Alteration in the Percentage of Layer 2/3 NF-Labeled Pyramidal Neurons Co-localized With H3K4me3, H3K27me3 or H3K27ac in APP/PS1 Mice Compared to Age-Matched Wild-Type Mice

**Figure 3:**
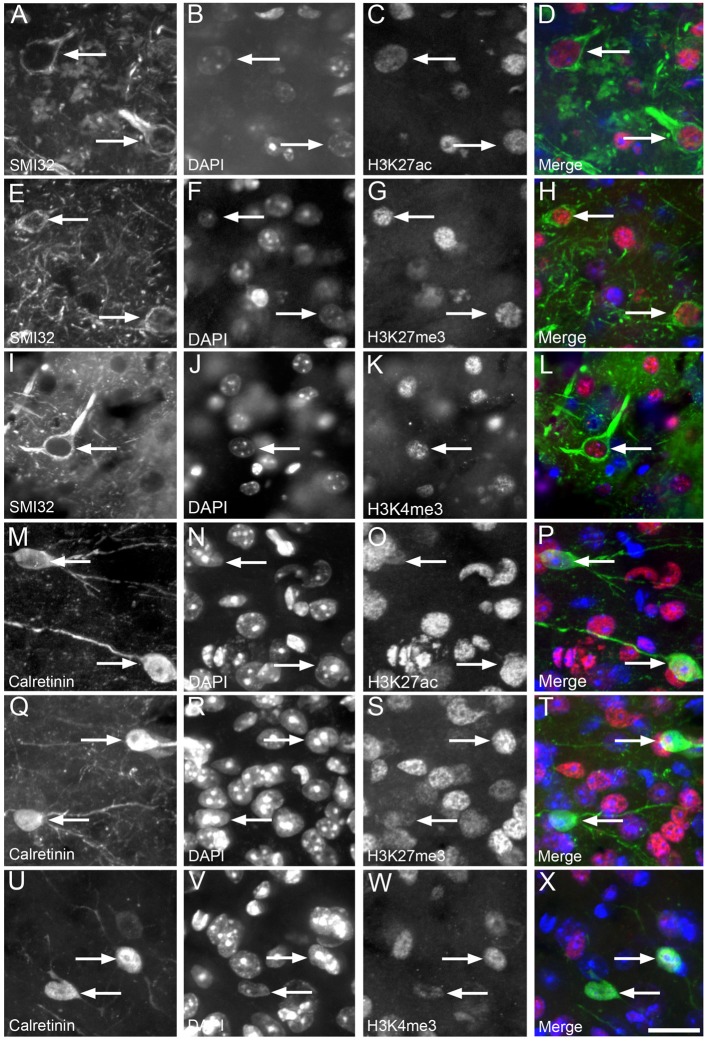
Nuclear H3K4me3, H3K27me3 and H3K27ac immunolabeling was observed in cortical layer 2/3 calretinin-positive interneurons and neurofilament (NF)-labeled pyramidal neurons. Representative images of layer 2/3 in a 12 month old WT mouse **(A–D)** showing NF-labeled pyramidal neurons (**A,D** green) co-localized (arrows) with DAPI staining (**B,D** blue) and H3K27ac immunoreactivity (**C,D** red). An example image of layer 2/3 in a 3 month old APP/PS1 mouse **(E–H)** showing NF-labeled pyramidal neurons (**E,H**, green) co-localized (arrows) with DAPI staining (**F,H**, blue) and H3K27me3 labeling (**G,H**, red). A representative image layer 2/3 in a 3 month old wild-type mouse **(I–L)** showing NF-labeled pyramidal neurons (**I,L**, green) co-localized (arrows) with DAPI (**J,L**, blue) and H3K4me3 immunoreactivity (**K,L**, red). An example image of layer 2/3 of a 3 month old APP/PS1 cortex **(M–P)** with calretinin-labeled interneurons (**M,P** green) co-localized (arrows) with DAPI staining (**N,P** blue) and H3K27ac immunoreactivity (**O,P** red). Representative image of layer 2/3 of a 3 month old APP/PS1 mouse **(Q–T)** illustrating calretinin-positive interneurons (**Q,T** green) containing (arrows) DAPI staining (**R,T** blue) and H3K27me3 labeling (**S,T** red). An example region of layer 2/3 of the cortex of a 6 month old wild-type mouse **(U–X)** showing calretinin-labeled interneurons (**U,X** green) co-localized (arrows) with DAPI staining (**V,X** blue) and H3K4me3 immunopositive nuclei (**W,X** red). Scale bar = 18 μm.

**Figure 4:**
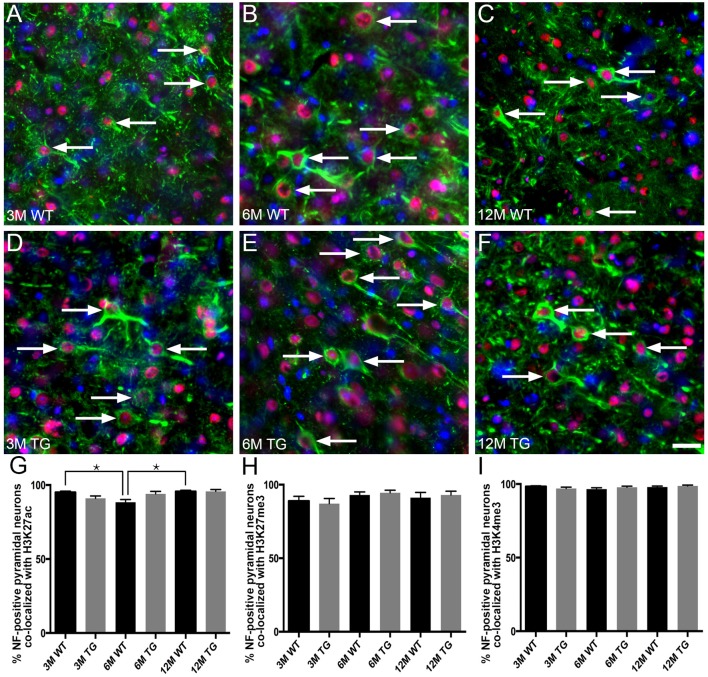
No difference in the percentage of layer 2/3 NF-labeled pyramidal neurons co-localized with H3K4me3, H3K27me3 or H3K27ac in APP/PS1 vs. wild-type mice. Representative images of layer 2/3 NF-positive pyramidal neurons (SMI-32, green) stained with DAPI (blue) and labeled for H3K27ac (red, arrows) in 3- **(A,D)**, 6- **(B,E)** and 12- **(C,F)** month-old wild-type **(A–C)** and APP/PS1 **(D–F)** mice. Bar graphs showing the percentage of NF-positive neuronal nuclei co-localized with H3K27ac **(G)**, H3K27me3 **(H)** and H3K4me3 **(I)** immunolabeling. All data are presented as mean ± SEM. ^*^p < 0.05. Scale bars: 25 μm.

H3K4me3, H3K27me3 and H3K27ac labeling were nuclear, with no cytoplasmic immunoreactivity observed either in NF-labeled pyramidal neurons or in calretinin-positive interneurons of cortical layer 2/3 (Figure 3). The vast majority (92%–98%) of NF-positive pyramidal neurons in layer 2/3 exhibited nuclear immunoreactivity for H3K27ac, H3K4me3 and H3K27me3 (Figure 4, Supplementary Table S1). There was a small (~7%), but significant decrease in the percentage of NF-labeled pyramidal neurons labeled with H3K27ac in 6-month-old wild-type mice compared to both 3- and 12-month-old wild-type mice (One Way ANOVA; *p* < 0.001; Figure 4G). There was no alteration in the percentage of NF-positive pyramidal neurons co-localized with H3K27ac, H3K4me3 or H3K27me3 in APP/PS1 mice compared to wild-type mice at 3, 6 and 12 months of age (H3K27ac, One Way ANOVA; H3K4me3 and H3K27me3, Kruskal-Wallis one way ANOVA on ranks; Figures 4G–I, Supplementary Figures S2, S3). Similarly, there was no difference between the proportion of NF-labeled pyramidal neurons that were immunoreactive for H3K4me3 or H3K27me3 in wild-type mice at 3, 6 and 12 months of age (Kruskal-Wallis one-way ANOVA on ranks; Figures 4H,I).

### No Change in the Proportion of Layer 2/3 Calretinin-Labeled Interneurons Co-localized With H3K27me3, H3K4me3 or H3K27ac in APP/PS1 Compared to Wild-Type Mice

**Figure 5:**
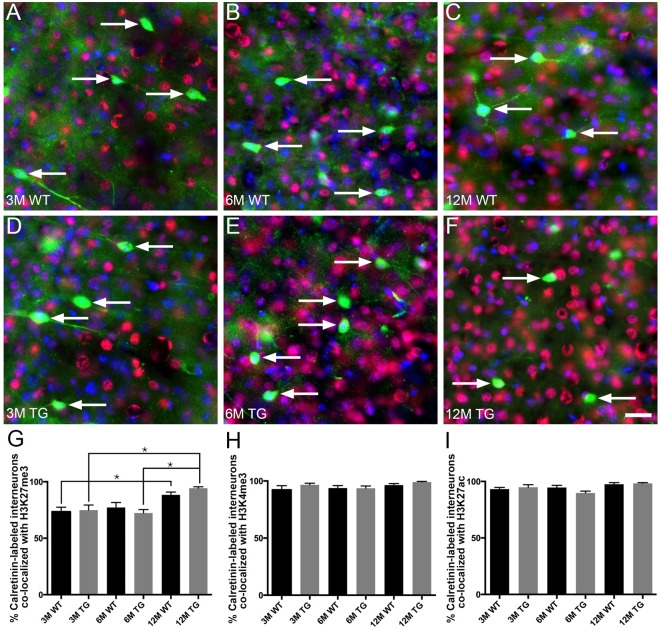
No difference in the percentage of layer 2/3 calretinin-positive interneurons co-localized with H3K27me3, H3K4me3 or H3K27ac in APP/PS1 vs. wild-type mice. Representative images of layer 2/3 calretinin-positive interneurons (green), stained with DAPI (blue) and labeled with H3K27me3 (red, arrows) in 3- **(A,D)**, 6- **(B,E)** and 12- **(C,F)** month-old wild-type **(A–C)** and APP/PS1 **(D–F)** mice. Bar graphs showing the percentage of calretinin-labeled interneurons co-localized with H3K27me3 **(G)**, H3K4me3 **(H)** and H3K27ac **(I)**. All data are presented as mean ± SEM. ^*^p < 0.05. Scale bars 25 μm.

The vast majority (~80%–95%) of layer 2/3 calretinin-positive interneurons were immunoreactive for H3K27me3, H3K4me3 or H3K27ac (Figure 5, Supplementary Table S1). Interestingly, there was an increase (~20%) in the percentage of calretinin-positive interneurons immunolabeled with H3K27me3 in 12-month-old APP/PS1 mice compared to both 3- and 6-month-old APP/PS1 mice (One Way ANOVA, *p* < 0.001; Figure 5G). There was no difference in the percentage of layer 2/3 calretinin-labeled interneurons co-localized with H3K27me3, H3K4me3 or H3K27ac between APP/PS1 and wild-type mice at 3, 6 and 12 months of age (H3K27me3, One way ANOVA; H3K4me3 and H3K27ac, Kruskal-Wallis one way ANOVA on ranks; Figures 5G–I, Supplementary Figures S4, S5). While previous work from our laboratory revealed that excitatory, but not inhibitory, presynaptic puncta are lost adjacent to Aβ plaques (Mitew et al., 2013a), we detected no difference in the density of presynaptic puncta containing synaptophysin (all presynaptic puncta), VGlut1 (excitatory presynaptic puncta) or VGAT (inhibitory presynaptic puncta) between 2-month-old and 12-month-old wild-type mice (Supplementary Figure S6, Table S1).

When NF-positive pyramidal neurons and calretinin-labeled interneurons were compared, a lower percentage of calretinin-labeled interneurons co-localized with H3K27me3 compared to NF-positive pyramidal neurons in 3- and 6-month old wild-type mice (*p* < 0.05, student’s *t*-test). There was no difference in the proportion of NF-immunoreactive pyramidal neurons compared to calretinin-labeled interneurons co-labeled with H3K4me3, H3K27ac in 3-, 6- and 12-month-old wild-type mice.

## Discussion

This is the first study to investigate the H3K4me3, H3K27ac and H3K27me3 histone marks in AD in specific subsets of neurons that are selectively vulnerable and resistant to AD pathology. We did not detect any global alterations in H3K4me3, H3K27ac or H3K27me3 across a time course in wild-type compared to APP/PS1 mice, in the nuclei of all cortical cell types, AD susceptible NF-positive pyramidal neurons or in AD-resilient calretinin-interneurons. Mastroeni et al. (2015) also detected no change in the overall protein level of H3K4me3 in the medial temporal gyrus of human AD cases compared to controls. However, the same study discovered abnormal H3K4me3 loss in the nucleus and gain of H3K4me3 in the cytoplasm in human AD cases compared to controls (Mastroeni et al., 2015). The mislocalized cytoplasmic H3K4me3 co-localized with pre-tangles and NFTs in late AD cases (Mastroeni et al., 2015). The authors also reported a loss of nuclear H3K4me3 in hippocampal subregions in the 3×Tg AD mouse model (which develop plaques and NFTs), but no cytoplasmic H3K4me3 immunoreactivity (Mastroeni et al., 2015). We did not observe any cytoplasmic accumulation or nuclear depletion of H3K4me3 in the cortex of APP/PS1 mice. These data suggest that H3K4me3 mislocalization may be region specific, and its occurrence in human AD, but not AD mouse models indicates that it could be a species or time-dependent process. It should be noted that APP/PS1 mice do not develop tau pathology, and so it is possible that the accumulation of abnormal tau and the deposition of NFTs may drive global changes and mislocalization of H3K4me3, H3K27ac and H3K27me3. Furthermore, this study only informs on the global level and subcellular localization of H3K4me3, H3K27ac and H3K427me3 and does not rule out robust global changes in other cell populations or at specific gene loci. Indeed, chromatin immunoprecipitation sequencing (ChIP-seq) data from AD cases and mouse models of neurodegeneration show that histone mark alterations certainly occur at specific loci in AD compared to control tissue (Gräff et al., 2012; Benito et al., 2015; Gjoneska et al., 2015; Nativio et al., 2018).

Our study captured age-related changes in histone marks in wild-type mice. There was a ~20% increase in the percentage of calretinin-labeled interneurons with H3K27me3 immunoreactivity at 12 months of age vs. 3 and 6 months of age, suggesting increased repression of the genome in calretinin-positive interneurons with aging. A previous study reported a decrease in H3K4me3 in whole brain homogenate in aged vs. young mice (Gong et al., 2015), while analysis of homogenate from rat hippocampal subregions showed increased H3K4me3 in the CA1 and CA3, but not in the dentate gyrus with age (Morse et al., 2015). Furthermore, aged (>60 years) human prefrontal cortex neurons exhibited a loss of H3K4me3 at 556 genes and a gain of H3K4me3 at 101 genes when compared to young (<1 year) neurons (Cheung et al., 2010). Taken together these data suggest that H3K4me3 likely alters across the course of healthy aging in a complex manner that is both region- and neuronal subtype-specific. The current study also detected a small but significant decrease (~7%) in the proportion of NF-positive pyramidal neurons co-localized with H3K27ac at 6 months of age compared to both 3 and 12 months of age. In keeping with our data, a study of whole brain homogenate did not detect any change in the overall level of H3K27ac between 3-month-old and 22-month-old mice (Gong et al., 2015). The physiological impact of a transient 15% decrease in the proportion of NF-labeled pyramidal neurons immunoreactive for H3K27ac at 6 months of age is currently unknown; the subset of neurons, genetic loci at which H3K27ac decreases and alterations in other epigenetic marks and modifiers would also need to be considered. It should also be noted that the interpretation of changes in H2K27ac levels may not be straightforward, with a recent study suggesting that genes that are up-regulated with age are characterized by a decrease in both activating H3K27ac at promoters and repressive H3K27ac within gene bodies (Cheng et al., 2018). Interestingly, no loss of presynaptic boutons (including excitatory or inhibitory subsets) was detected in 12-month-old wild-type compared to 2-month-old wild-type mice in the current study. These data, together with previous work demonstrating plaque-associated synapse loss in 10–12-month-old APP/PS1 mice (Mitew et al., 2013a), suggest that the global changes in H3K27me3, H3K27ac and H3K4me3 levels in NF-labeled pyramidal neurons or calretinin-positive interneurons do not correlate with synapse loss in APP/PS1 or wild-type mice.

This study also identified inherent differences in H3K27me3 expression between calretinin-labeled interneurons and NF-immunoreactive pyramidal neurons. The lower expression of H3K27me3 in calretinin-positive interneurons suggesting a less repressed epigenome in this interneuron subpopulation. Several studies have documented distinct epigenetic profiles for excitatory and inhibitory neurons to date (Mo et al., 2015; Kozlenkov et al., 2016; Luo et al., 2017; Gasparoni et al., 2018). In keeping with our data, DNA methylation data in human cortex also identified decreased methylation of CpG sites in inhibitory neurons compared to excitatory neurons (Kozlenkov et al., 2016). However, excitatory neurons from mouse exhibited increased chromatin accessibility and increased DNA hypomethylation compared to different murine inhibitory interneuron subtypes (parvalbumin- and vasoactive intestinal peptide-interneurons; Mo et al., 2015). We have also previously detected differences in 5mC and 5hmC labeling in NF-positive pyramidal neurons and calretinin-immunoreactive interneurons in the human brain (Phipps et al., 2016). Differences in the epigenetic landscape of neuronal subtypes are to be expected, especially considering the vast diversity of neuronal function and plasticity in the brain. In fact, recent *t*-SNE analysis of single-neuron DNA methylation data have predicted that there are 16 distinct subpopulations of neurons in the frontal cortex of the mouse and 21 in the human frontal cortex (Luo et al., 2017), indicating more extensive functional diversity and network complexity in the cortex than had previously been predicted.

The use of the APP/P1 mouse model is a limitation of the current study as it does not develop the neurofibrillary pathology or overt cell loss that occurs in human AD. Thus, we cannot rule out the possibility that global levels of H3K4me3, H3K27ac or H2K37me3 may alter in disease-resistant and–vulnerable subpopulations of neurons in the presence of tau pathology. Indeed, Mastroeni et al. (2015) described abnormal translocation of H3K4me3 from the nucleus to the cytoplasm that correlated with tau pathology load in the middle temporal gyrus of AD cases. Recent research also reported that tau load, but not Aβ load, correlated with H3K9ac marking in the prefrontal cortex in AD and demonstrated that tau overexpression alters spatial chromatin structure (Klein et al., 2019). Furthermore, the current study uses immunohistochemistry, and while this technique can provide valuable information regarding global levels of histone marks and their intracellular location in subtypes of neurons it does not provide base pair level resolution of histone mark alterations across the genome.

## Conclusion

We present data, for the first time, describing no global alterations in the proportion of AD-susceptible NF-labeled pyramidal neurons or AD-resistant calretinin-positive interneurons co-localized with the H3K4me3, H3K27ac and H3K27me3 histone marks across a time course in APP/PS1 and wild-type mice. We report alterations in the global levels of H3K27ac in NF-positive pyramidal neurons and in H3K27me3 in calretinin-immunoreactive interneurons with age. Our data suggest that amyloid deposition does not impact the global levels of H3K4me3, H3K27ac and H3K27me3 in the two neuronal populations and at the time points examined in this study. However, it is important to note that the global levels of histone marks can remain unaltered concomitant with dramatic changes at specific loci; as alterations of histone marks at specific gene loci certainly occurs in AD (Benito et al., 2015; Gjoneska et al., 2015; Nativio et al., 2018).

## Data Availability

The raw data supporting the conclusions of this manuscript will be made available by the authors, without undue reservation, to any qualified researcher.

## Author Contributions

AW and PT designed the experiments. MD, AP and SM performed the experiments. MD and SM performed the analysis. All authors contributed to manuscript preparation. All authors read and approved the final manuscript.

## Conflict of Interest Statement

The authors declare that the research was conducted in the absence of any commercial or financial relationships that could be construed as a potential conflict of interest.
