# Supplementary figures and images for: Age, but Not Amyloidosis, Induced Changes in Global Levels of Histone Modifications in Susceptible and Disease-Resistant Neurons in Alzheimer’s Disease Model Mice
Source: Front Aging Neurosci. 2019 Apr 3;11:68. doi: 10.3389/fnagi.2019.00068 (PMC6456813; doi:10.3389/fnagi.2019.00068)

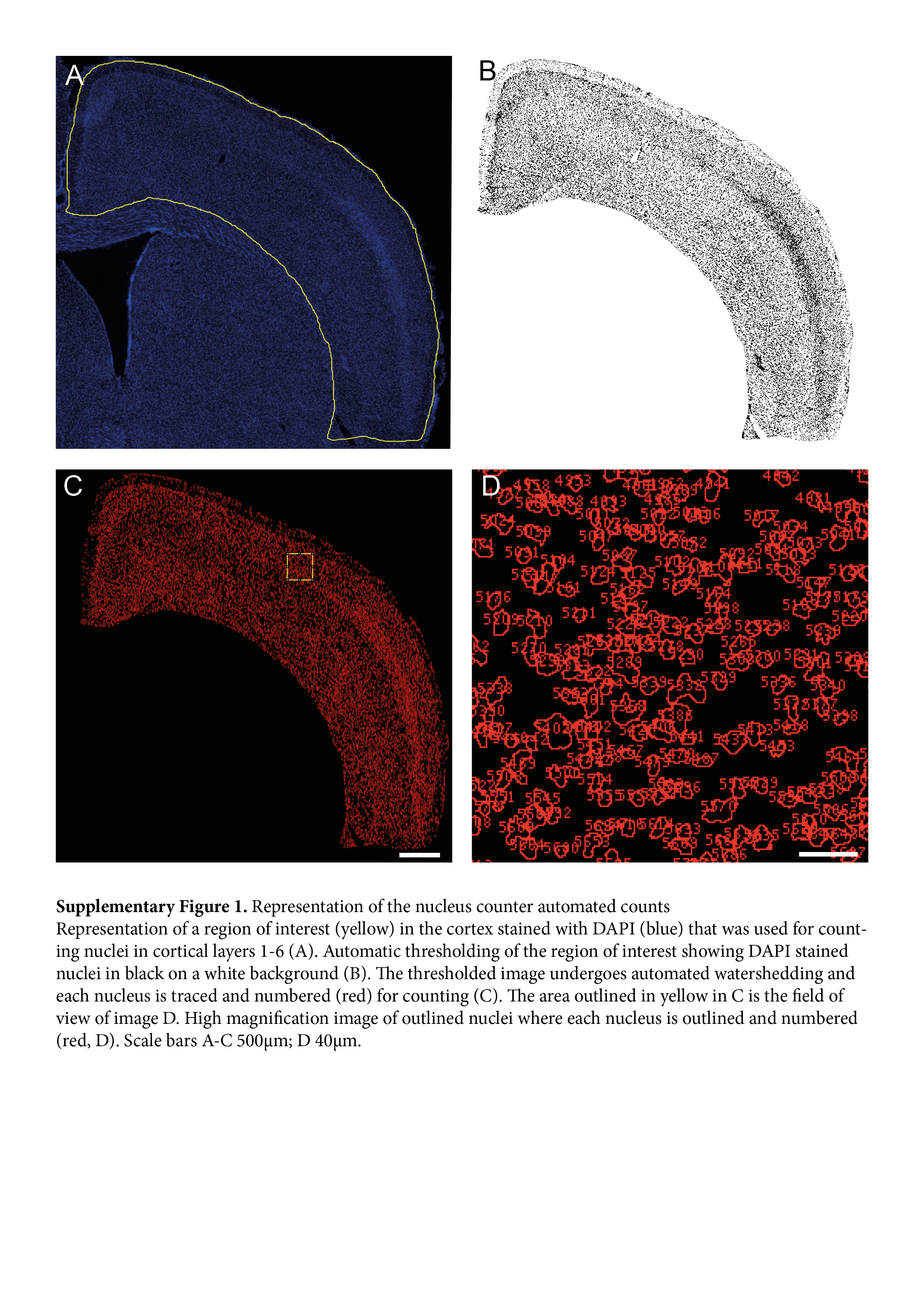

Supplement: Supplementary file 1 [file Image_1.JPEG]

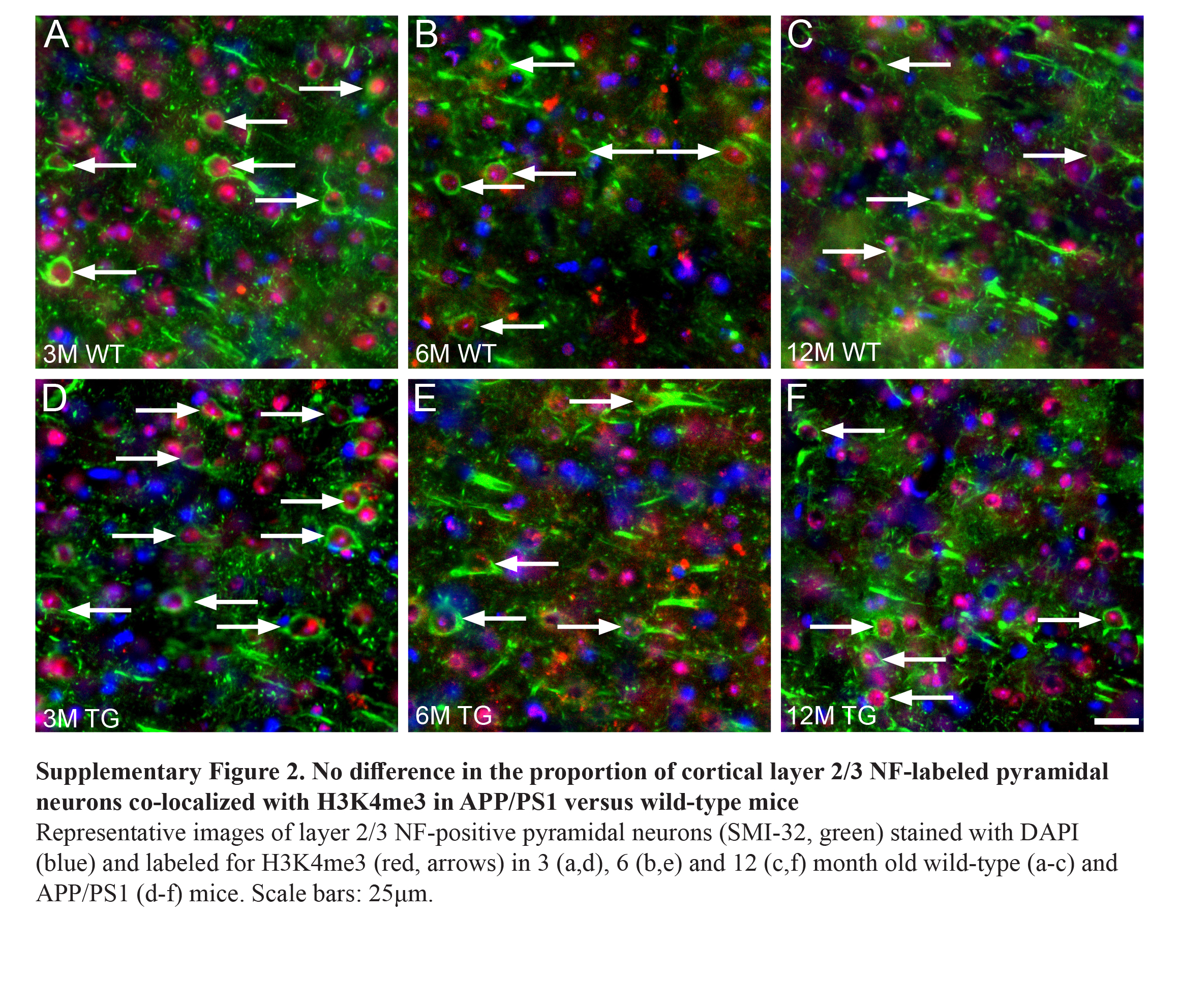

Supplement: Supplementary file 2 [file Image_2.JPEG]

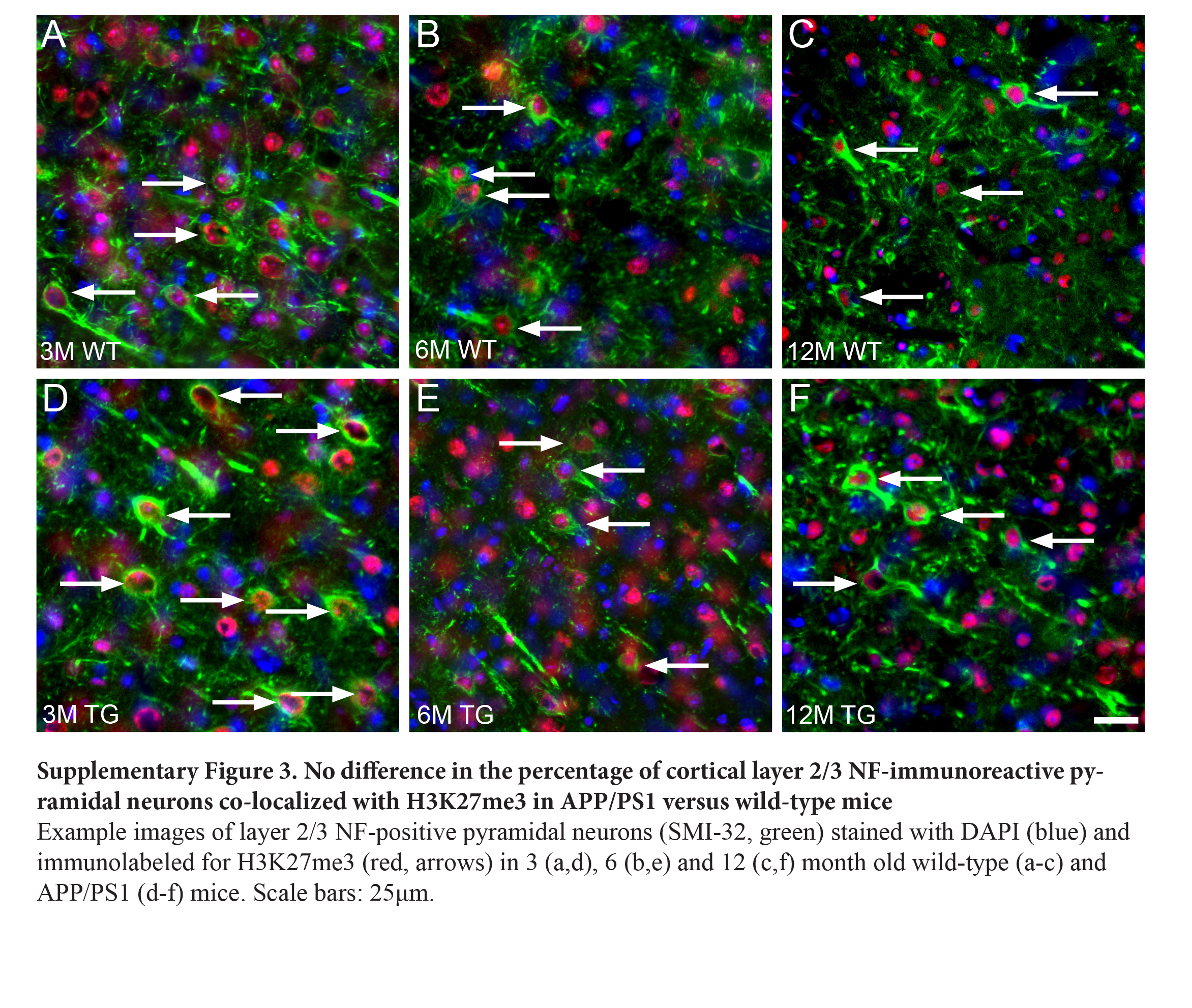

Supplement: Supplementary file 3 [file Image_3.JPEG]

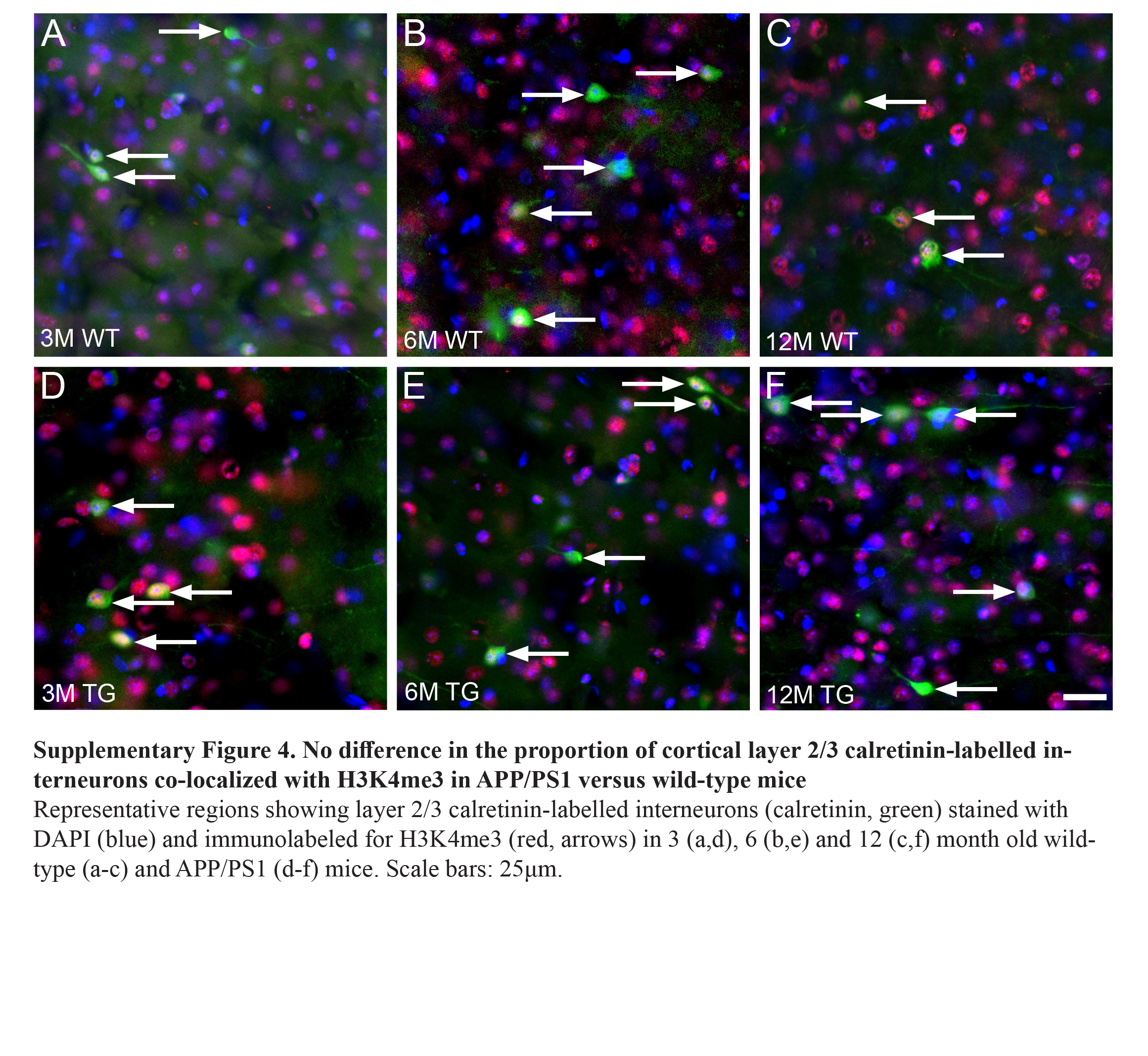

Supplement: Supplementary file 4 [file Image_4.JPEG]

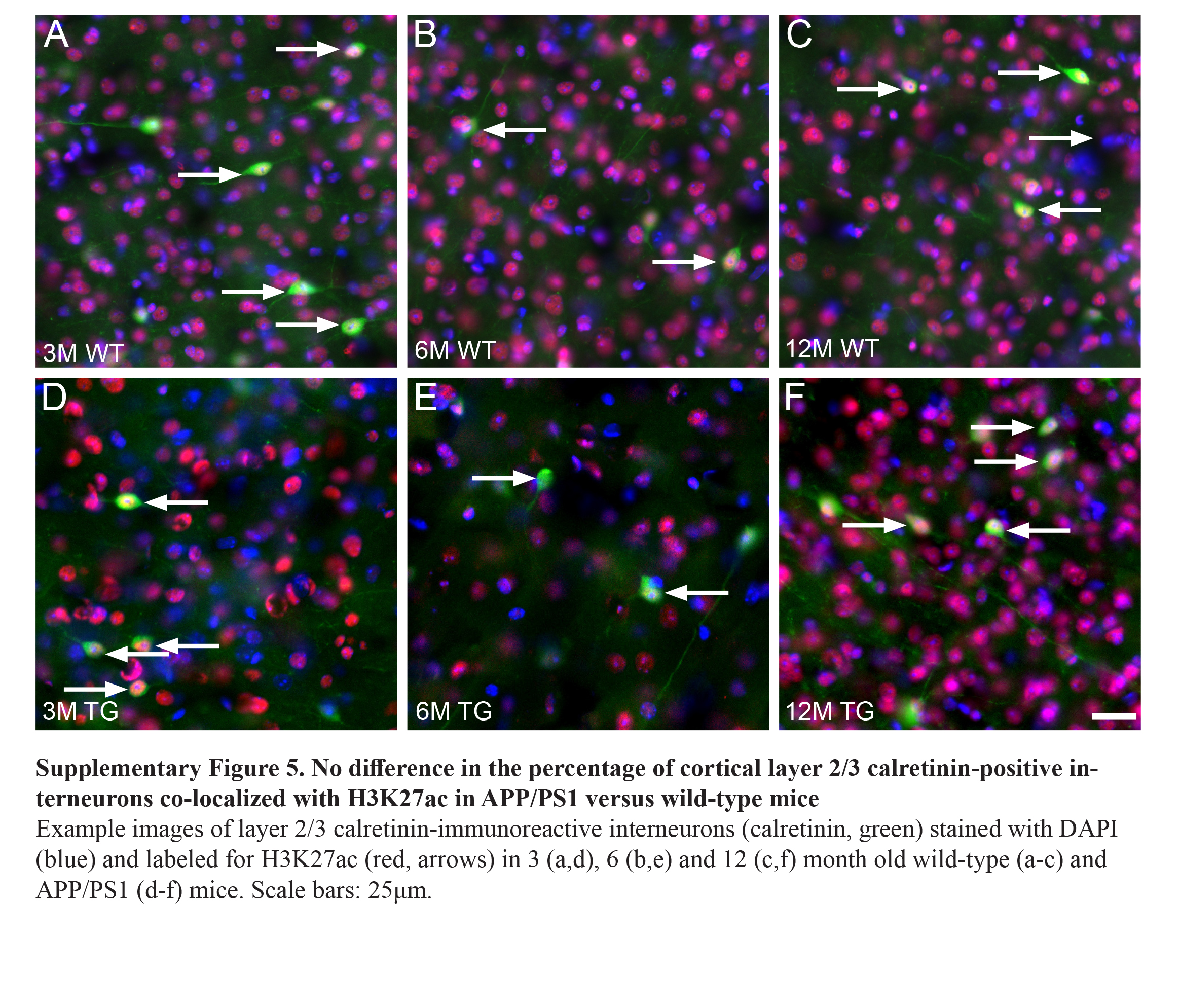

Supplement: Supplementary file 5 [file Image_5.JPEG]

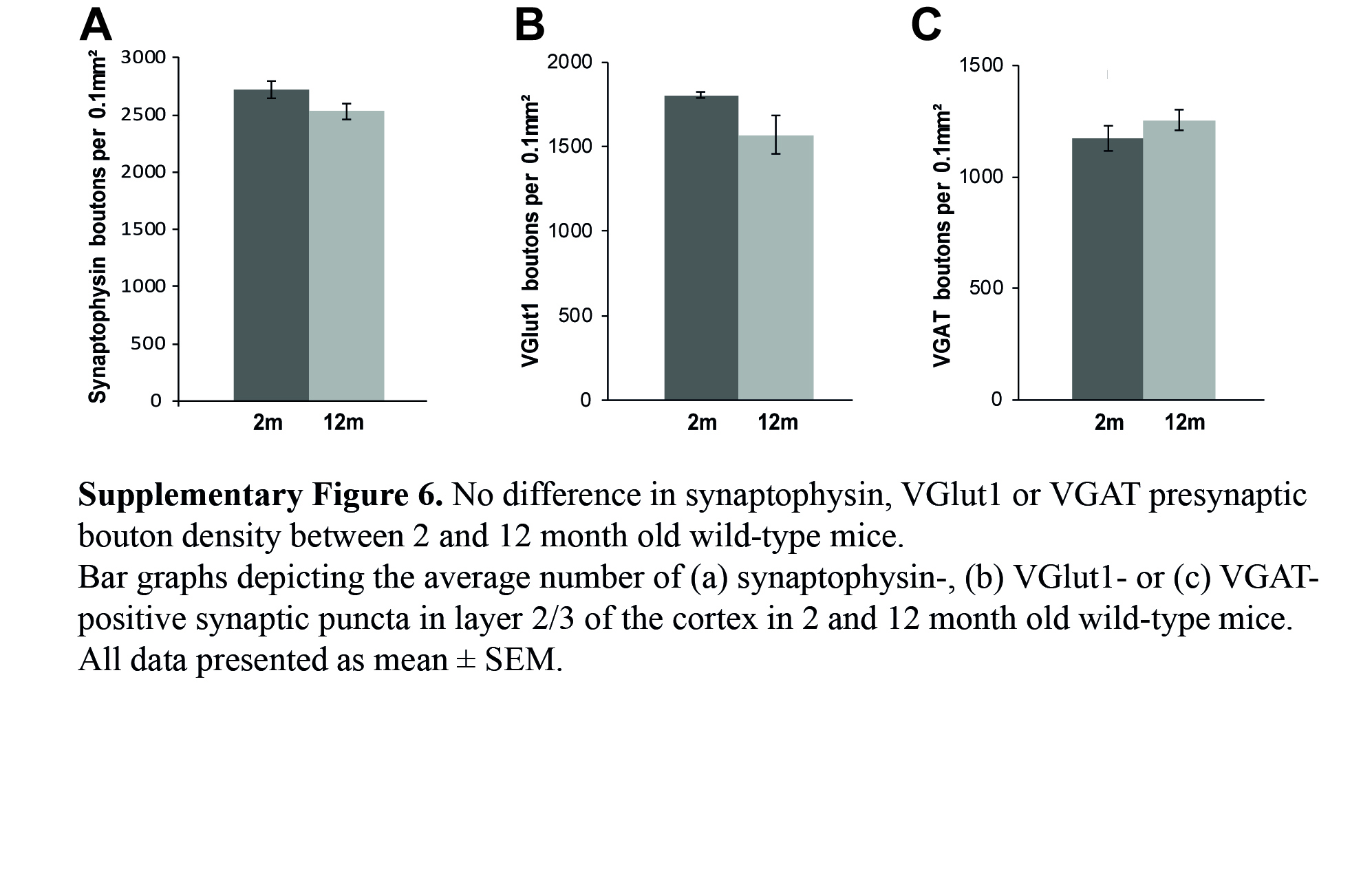

Supplement: Supplementary file 6 [file Image_6.JPEG]
