# Supplementary material for: Age, but Not Amyloidosis, Induced Changes in Global Levels of Histone Modifications in Susceptible and Disease-Resistant Neurons in Alzheimer’s Disease Model Mice
Source: Front Aging Neurosci. 2019 Apr 3;11:68. doi: 10.3389/fnagi.2019.00068 (PMC6456813; doi:10.3389/fnagi.2019.00068)
Supplement: Supplementary file 7 [file Data_Sheet_1.docx]

**Supplementary Material**

**Supplementary Materials and Methods**

***Primary antibody specificity***

*HK4me3:* The H3K4me3 primary antibody (Table 1) is modENCODE validated and NG-S-QC certified for next generation sequencing studies, it labels one band (~20kDa) when nuclear extract is run on a Western blot, and dot blot analysis shows that it does not cross-react with unmodified H3K4, H3K4me1, H3K4me2, unmodified H3K9, H3K9me1, H3K9me2, H3K9me3, unmodified H3K79, H3K79me1, H3K79me2, H3K79me3, unmodified H3K23, H3K23me1, H3K23me3, H3K23me3, unmodified H3K27, H3K27me1, H3K27me2, H3K27me3, unmodified H3K36, H3K36me1, H3K36me2, or H3K36me3 (manufacturer information).

*HK27ac:* The H3K27ac primary antibody (Table 1) is modENCODE validated and NG-S-QC certified for next generation sequencing studies, it labels one band (~17kDa) when nuclear extract is run on a Western blot, and dot blot analysis shows that it does not cross-react with H3K37ac, Unmodified H3K37, H3K36ac, H3K9ac, H3K14ac, H3K18ac, H3K23ac, H4K5ac, H4K8ac, H4K12ac or H4K16ac (manufacturer information).

*H3K27me3:* The H3K27me3 primary antibody (Table 1) is widely used for next-generation sequencing studies (Taberlay et al., 2011; Tao et al., 2019), labels a single band (~18kDa) on Western blot and dot blot analysis shows that it does not cross-react with a wide range similar epitopes including unmodified H3, H3K4me1, H3K4me2, H3K3me3, H3K9ac, H3K9me1, H3K9me2, H3K14ac, H3K18ac, H3K23ac, H3K27ac, H3K27me1, H3K27me2, H3K36ac, H3K36me1, H3K36me2, H3K36me3, H3K56ac, H3K79me1, H3K79me2 and H3K79me3 (manufacturer information).

*SMI-32:* The SMI-32 primary antibody (Table 1) recognises the neurofilament-medium and neurofilament-high subunits it has been widely used to label NF-positive pyramidal neurons (Woodhouse et al., 2009a; Woodhouse et al., 2009b; Mitew et al., 2013b; Fernandez-Martos et al., 2015) and it labels two bands on Western blot (~160kDa and ~200kDa) corresponding to the NFM and NFH subunits (manufacturer information).

*Calretinin:* The calretinin primary antibody (Table 1) labels a single ~29kDa band on Western blot corresponding to calretinin, furthermore, the calretinin primary antibody labels a subpopulation of neurons in wild-type mouse brain but does not label the brain of calretinin knock out mice (manufacturer information).

*6E10:* The 6E10 antibody is widely used in Alzheimer’s research and recognises both Aβ and the amyloid precursor protein. Western blot analysis shows that 6E10 labelled 2 bands in 9 month old APP/PS1 mice at ~10kDa and ~110kDa, and no 6E10-labelled bands were present brain homogenate from 9 month old wild-type mice (Collins et al., 2015).

*Synaptophysin:* The synaptophysin primary antibody (Table 1) has been widely used to analyse presynaptic puncta (Mitew et al., 2013a; Collins et al., 2015; Fernandez-Martos et al., 2015; Stuart et al., 2016) and it labels a single band at ~38kDA on Western blots (Stuart and manufacturer information).

*VGlut1:* The VGlut1 primary antibody (Table 1) labels VGlut1-positive synaptic boutons in wild-type mice but no immunosignal is present in VGlut1 knock out mice (manufacturer information), it labels a band at ~61kDa corresponding to VGlut1 in Western Blot (Hayashi et al., 2016; Vannini et al., 2016; Trobiani et al., 2018).

*VGAT:* The VGAT primary antibody (Table 1) labels VGAT-containing synaptic puncta in wild-type mice but no immunosignal is present in VGAT knock out mice (manufacturer information), the VGAT primary antibody labels a band at ~56kDa on Western blot (Li et al., 2018; Seigneur and Sudhof, 2018).

**Supplementary Table 1: Data Summary**

|  | **Wild-type** | **APP/PS1** |
| --- | --- | --- |
| % all cortical nuclei layers 1-6 co-localized with H3K4me3, 3 months | 83.0 ± 2.2 | 78.1 ± 3.8 |
| % all cortical nuclei layers 1-6 co-localized with H3K4me3, 6 months | 83.1 ± 1.2 | 78.4 ± 3.2 |
| % all cortical nuclei layers 1-6 co-localized with H3K4me3, 12 months | 71.9 ± 8.3 | 84.8 ± 1.8 |
| % all cortical nuclei layers 1-6 co-localized with H3K27ac, 3 months | 77.8 ± 2.6 | 76.6 ± 5.1 |
| % all cortical nuclei layers 1-6 co-localized with H3K27ac, 6 months | 79.9 ± 4.0 | 77.6 ± 3.1 |
| % all cortical nuclei layers 1-6 co-localized with H3K27ac, 12 months | 78.8 ± 3.7 | 74.7 ± 3.3 |
| % all cortical nuclei layers 1-6 co-localized with H3K27me3, 3 months | 64.7 ± 2.7 | 66.9 ± 1.5 |
| % all cortical nuclei layers 1-6 co-localized with H3K27me3, 6 months | 67.4 ± 3.6 | 69.8 ± 4.0 |
| % all cortical nuclei layers 1-6 co-localized with H3K27me3, 12 months | 67.5 ± 3.1 | 65.4 ± 3.5 |
| % all cortical nuclei layer 2/3 co-localized with H3K4me3, 3 months | 84.8 ± 2.7 | 78.6 ± 4.6 |
| % all cortical nuclei layer 2/3 co-localized with H3K4me3, 6 months | 75.5 ± 5.6 | 74.2 ± 4.2 |
| % all cortical nuclei layer 2/3 co-localized with H3K4me3, 12 months | 72.6 ± 5.1 | 83.6 ± 2.8 |
| % all cortical nuclei layer 2/3 co-localized with H3K27ac, 3 months | 87.8 ± 2.4 | 84.0 ± 2.5 |
| % all cortical nuclei layer 2/3 co-localized with H3K27ac, 6 months | 84.2 ± 3.1 | 80.0 ± 3.2 |
| % all cortical nuclei layer 2/3 co-localized with H3K27ac, 12 months | 80.8 ± 3.8 | 79.7 ± 3.4 |
| % all cortical nuclei layer 2/3 co-localized with H3K27me3, 3 months | 70.6 ± 3.0 | 74.1 ± 3.5 |
| % all cortical nuclei layer 2/3 co-localized with H3K27me3, 6 months | 74.4 ± 5.4 | 69.2 ± 2.8 |
| % all cortical nuclei layer 2/3 co-localized with H3K27me3, 12 months | 75.2 ± 3.9 | 71.5 ± 4.5 |
| % NF-labeled pyramidal neurons co-localized with H3K4me3, 3 months | 98.2 ± 1.5 | 97.3 ± 2.6 |
| % NF-labeled pyramidal neurons co-localized with H3K4me3, 6 months | 96.0 ± 1.1 | 97.8 ± 0.7 |
| % NF-labeled pyramidal neurons co-localized with H3K4me3, 12 months | 98.0 ± 0.9 | 99.3 ± 0.4 |
| % NF-labeled pyramidal neurons co-localized with H3K27ac, 3 months | 95.0 ± 0.9 | 91.0 ± 1.6 |
| % NF-labeled pyramidal neurons co-localized with H3K27ac, 6 months | 88.4 ± 1.8 | 94.0 ± 1.7 |
| % NF-labeled pyramidal neurons co-localized with H3K27ac, 12 months | 95.6 ± 0.9 | 95.7 ± 1.3 |
| % NF-labeled pyramidal neurons co-localized with H3K27me3, 3 months | 88.5 ± 3.6 | 90.2 ± 2.6 |
| % NF-labeled pyramidal neurons co-localized with H3K27me3, 6 months | 92.7 ± 2.3 | 94.8 ± 1.7 |
| % NF-labeled pyramidal neurons co-localized with H3K27me3, 12 months | 96.0 ± 2.2 | 92.4 ± 3.1 |
| % calretinin-positive interneurons co-localized with H3K4me3, 3 months | 92.2 ± 3.7 | 96.7 ± 1.4 |
| % calretinin-positive interneurons co-localized with H3K4me3, 6 months | 93.8 ± 2.1 | 93.6 ± 2.1 |
| % calretinin-positive interneurons co-localized with H3K4me3, 12 months | 96.4 ± 1.3 | 99.0 ± 0.6 |
| % calretinin-positive interneurons co-localized with H3K27ac, 3 months | 93.2 ± 1.5 | 94.9 ± 2.2 |
| % calretinin-positive interneurons co-localized with H3K27ac, 6 months | 94.7 ± 1.8 | 89.8 ± 1.7 |
| % calretinin-positive interneurons co-localized with H3K27ac, 12 months | 97.6 ± 1.4 | 98.3 ± 0.7 |
| % calretinin-positive interneurons co-localized with H3K27me3, 3 months | 73.4 ± 4.0 | 74.9 ± 4.5 |
| % calretinin-positive interneurons co-localized with H3K27me3, 6 months | 77.2 ± 4.5 | 72.2 ± 3.1 |
| % calretinin-positive interneurons co-localized with H3K27me3, 12 months | 88.4 ± 2.5 | 94.3 ± 1.3 |
| Synaptophysin-positive puncta per 0.1mm^2^, 2 months | 2722 ± 77 |  |
| Synaptophysin-positive puncta per 0.1mm^2^, 12 months | 2530 ± 69 |  |
| Vesicular glutamate transporter-positive puncta per 0.1mm^2^, 2 months | 1804 ± 24 |  |
| Vesicular glutamate transporter-positive puncta per 0.1mm^2^, 12 months | 1570 ± 111 |  |
| Vesicular GABA transporter-positive puncta per 0.1mm^2^, 2 months | 1172 ± 155 |  |
| Vesicular GABA transporter-positive puncta per 0.1mm^2^, 12 months | 1253 ± 47 |  |

***References:***

Collins, J.M., King, A.E., Woodhouse, A., Kirkcaldie, M.T., and Vickers, J.C. (2015). The effect of focal brain injury on beta-amyloid plaque deposition, inflammation and synapses in the APP/PS1 mouse model of Alzheimer's disease. *Exp Neurol* 267**,** 219-229. doi: 10.1016/j.expneurol.2015.02.034.

Fernandez-Martos, C.M., King, A.E., Atkinson, R.A., Woodhouse, A., and Vickers, J.C. (2015). Neurofilament light gene deletion exacerbates amyloid, dystrophic neurite, and synaptic pathology in the APP/PS1 transgenic model of Alzheimer's disease. *Neurobiol Aging* 36(10)**,** 2757-2767. doi: 10.1016/j.neurobiolaging.2015.07.003.

Hayashi, Y., Nishimune, H., Hozumi, K., Saga, Y., Harada, A., Yuzaki, M., et al. (2016). A novel non-canonical Notch signaling regulates expression of synaptic vesicle proteins in excitatory neurons. *Sci Rep* 6**,** 23969. doi: 10.1038/srep23969.

Li, Y., Kim, R., Cho, Y.S., Song, W.S., Kim, D., Kim, K., et al. (2018). Lrfn2-Mutant Mice Display Suppressed Synaptic Plasticity and Inhibitory Synapse Development and Abnormal Social Communication and Startle Response. *J Neurosci* 38(26)**,** 5872-5887. doi: 10.1523/jneurosci.3321-17.2018.

Mitew, S., Kirkcaldie, M.T., Dickson, T.C., and Vickers, J.C. (2013a). Altered synapses and gliotransmission in Alzheimer's disease and AD model mice. *Neurobiol Aging* 34(10)**,** 2341-2351. doi: 10.1016/j.neurobiolaging.2013.04.010.

Mitew, S., Kirkcaldie, M.T., Dickson, T.C., and Vickers, J.C. (2013b). Neurites containing the neurofilament-triplet proteins are selectively vulnerable to cytoskeletal pathology in Alzheimer's disease and transgenic mouse models. *Front Neuroanat* 7**,** 30. doi: 10.3389/fnana.2013.00030.

Seigneur, E., and Sudhof, T.C. (2018). Genetic Ablation of All Cerebellins Reveals Synapse Organizer Functions in Multiple Regions Throughout the Brain. *J Neurosci* 38(20)**,** 4774-4790. doi: 10.1523/jneurosci.0360-18.2018.

Stuart, K.E., King, A.E., Fernandez-Martos, C.M., Dittmann, J., Summers, M.J., and Vickers, J.C. (2016). Mid-life environmental enrichment increases synaptic density in CA1 in a mouse model of Abeta-associated pathology and positively influences synaptic and cognitive health in healthy ageing. *J Comp Neurol*. doi: 10.1002/cne.24156.

Taberlay, P.C., Kelly, T.K., Liu, C.C., You, J.S., De Carvalho, D.D., Miranda, T.B., et al. (2011). Polycomb-repressed genes have permissive enhancers that initiate reprogramming. *Cell* 147(6)**,** 1283-1294. doi: 10.1016/j.cell.2011.10.040.

Tao, Y., Kang, B., Petkovich, D.A., Bhandari, Y.R., In, J., Stein-O'Brien, G., et al. (2019). Aging-like Spontaneous Epigenetic Silencing Facilitates Wnt Activation, Stemness, and Braf(V600E)-Induced Tumorigenesis. *Cancer Cell* 35(2)**,** 315-328.e316. doi: 10.1016/j.ccell.2019.01.005.

Trobiani, L., Favaloro, F.L., Di Castro, M.A., Di Mattia, M., Cariello, M., Miranda, E., et al. (2018). UPR activation specifically modulates glutamate neurotransmission in the cerebellum of a mouse model of autism. *Neurobiol Dis* 120**,** 139-150. doi: 10.1016/j.nbd.2018.08.026.

Vannini, E., Restani, L., Pietrasanta, M., Panarese, A., Mazzoni, A., Rossetto, O., et al. (2016). Altered sensory processing and dendritic remodeling in hyperexcitable visual cortical networks. *Brain Struct Funct* 221(6)**,** 2919-2936. doi: 10.1007/s00429-015-1080-1.

Woodhouse, A., Shepherd, C.E., Sokolova, A., Carroll, V.L., King, A.E., Halliday, G.M., et al. (2009a). Cytoskeletal alterations differentiate presenilin-1 and sporadic Alzheimer's disease. *Acta Neuropathol* 117(1)**,** 19-29. doi: 10.1007/s00401-008-0458-z.

Woodhouse, A., Vickers, J.C., Adlard, P.A., and Dickson, T.C. (2009b). Dystrophic neurites in TgCRND8 and Tg2576 mice mimic human pathological brain aging. *Neurobiol Aging* 30(6)**,** 864-874. doi: 10.1016/j.neurobiolaging.2007.09.003.
